# Age Prediction of Hematoma from Hyperspectral Images Using Convolutional Neural Networks

**DOI:** 10.3390/jimaging12020078

**Published:** 2026-02-11

**Authors:** Arash Keshavarz, Gerald Bieber, Daniel Wulff, Carsten Babian, Stefan Lüdtke

**Affiliations:** 1 Visual Computing, Fraunhofer-Institute for Computer Graphics Research IGD, 18059 Rostock, Germany; 2 Institute of Visual & Analytic Computing, University of Rostock, 18059 Rostock, Germany; 3 Institute of Forensic Medicine Leipzig, 04103 Leipzig, Germany

**Keywords:** hyperspectral imaging, hematoma evolution, deep learning, spectral–spatial modeling, optical sensors, biomedical imaging

## Abstract

Accurate estimation of hematoma age remains a major challenge in forensic practice, as current assessments rely heavily on subjective visual interpretation. Hyperspectral imaging (HSI) captures rich spectral signatures that may reflect the biochemical evolution of hematomas over time. This study evaluates whether a convolutional neural network (CNN) integrating both spectral and spatial information improves hematoma age estimation accuracy. Additionally, we investigate whether performance can be maintained using a reduced, physiologically motivated subset of wavelengths. Using a dataset of forearm hematomas from 25 participants, we applied radiometric normalization and SAM-based segmentation to extract 64×64×204 hyperspectral patches. In leave-one-subject-out cross-validation, the CNN outperformed a spectral-only Lasso baseline, reducing the mean absolute error (MAE) from 3.24 days to 2.29 days. Band-importance analysis combining SmoothGrad and occlusion sensitivity identified 20 highly informative wavelengths; using only these bands matched or exceeded the accuracy of the full 204-band model across early, middle, and late hematoma stages. These results demonstrate that spectral–spatial modeling and physiologically grounded band selection can enhance estimation accuracy while significantly reducing data dimensionality. This approach supports the development of compact multispectral systems for objective clinical and forensic evaluation.

## 1. Introduction

Hematomas are localized collections of blood within the tissue outside blood vessels [1], typically resulting from blunt trauma that causes leakage into surrounding tissues. The assessment of hematoma plays a crucial role in various clinical and forensic contexts. In forensic medicine, estimating hematoma age can provide valuable information for narrowing down the time of injury and reconstructing the sequence of events leading to trauma [2].

In current forensic practice, hematoma age estimation is predominantly based on visual inspection of external characteristics such as color and consistency. However, it has been shown that the subjective visual assessment of hematoma age based on photographs is unreliable, and the accuracy of the results does not increase with a higher degree of forensic expertise [3,4]. This subjectivity highlights the need for objective, reproducible, and non-invasive methods for hematoma age assessment.

Early work on hematoma assessment focused on reflectance spectrophotometry, which quantifies color changes in the visible spectrum associated with hemoglobin degradation products [5,6]. Subsequent approaches attempted to estimate bruise age by matching measured spectra to simulated absorption models [7]. More recently, bio-optical techniques targeting depth information have been explored; for instance, pulsed photothermal radiometric depth profiling has been proposed to estimate the depth distribution of bruises by exploiting wavelength-dependent light penetration [8]. Such techniques explicitly address the three-dimensional structure of hematomas.

Despite these technological advances, recent work has shown that the practical adoption of different optical modalities varies substantially. Zimmermann et al. [9] compared smartphone imaging, Diffuse Reflectance Spectroscopy (DRS), and Hyperspectral Imaging (HSI), demonstrating that while smartphone-based imaging offers superior accessibility, it lacks the spectral richness required to capture subtle biochemical changes in tissue. In contrast, HSI provides spatially resolved spectral information that cannot be obtained from standard RGB images. Nevertheless, most deep learning-based approaches for hematoma age estimation have predominantly relied on RGB photographs and formulated the task as a classification problem [10]. This highlights a disconnect between the rich information provided by advanced optical modalities and the limited data representations commonly used in learning-based age estimation methods.

To bridge this gap, we look to the broader field of biomedical optics, where HSI has emerged as a powerful modality offering dense spectral sampling while preserving spatial information. Systematic and literature reviews have highlighted HSI as a promising tool for non-invasive perfusion assessment in clinical settings [11,12,13]. These reviews emphasize the ability of HSI to derive physiologically meaningful parameters, such as tissue oxygen saturation, hemoglobin concentration, and perfusion-related indices, from spatially resolved spectral data. Unlike RGB imaging, HSI allows the analysis of spectral signatures from both superficial tissues [14] and deeper structures, such as parenchymal tissue during liver radiofrequency ablation [15]. Specific to oncological settings, recent overviews describe how HSI has demonstrated potential as an intraoperative guidance tool by characterizing blood flow changes and oxygenation maps [11,12,16]. Furthermore, it has been utilized to quantify circulation deficits in flap monitoring [17] and peripheral arterial disease [18,19].

These advances directly motivate the application of HSI to objective hematoma age assessment. A recent study by Al-Arami et al. [20] demonstrated that hematoma age prediction is feasible using HSI data by averaging the spectral reflectance across manually segmented hematoma regions. While their results were encouraging, the approach relied on manual segmentation and exclusively spectral features, limiting scalability and neglecting spatial heterogeneity within the hematoma.

In this paper, we propose a semi-automatic pipeline for hematoma age estimation from hyperspectral images. Our approach leverages a convolutional neural network (CNN) to learn spectral–spatial features directly from the data. This study has the following main objectives:

- To develop a CNN-based model for hematoma age estimation and demonstrate its accuracy improvement over baseline networks.
- To evaluate the model’s robustness and generalization capability across all hematoma developmental stages.
- To determine the most informative wavelength regions, enabling reduced spectral dimensionality and computational complexity without compromising accuracy.

## 2. Materials and Methods

### 2.1. Dataset

**Figure 1:**
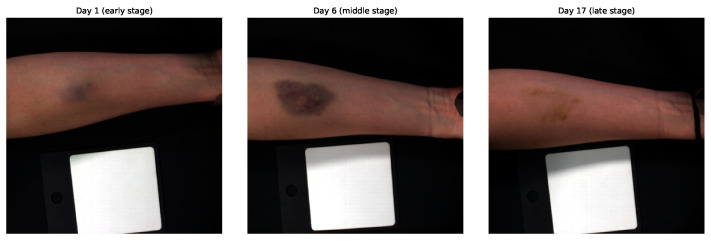
Representative hyperspectral RGB composites from early (Day 1), middle (Day 6), and late (Day 17) hematoma stages for one participant. These images illustrate the temporal evolution of hematoma appearance over the 21-day observation period.

This study utilized the HSI dataset described by Al-Arami et al. [20]. The dataset includes images from 25 healthy participants (15 male, 10 female) aged between 20 and 40 years. For each participant, a standardized hematoma was induced by injecting 3 mL of autologous blood into the volar side of the forearm. Hyperspectral images were acquired using the Specim IQ^®^ camera (Specim, Spectral Imaging Ltd., Oulu, Finland). Imaging was performed immediately before and after hematoma induction, followed by daily acquisitions during the first seven days and then every 48 h over the subsequent two weeks, resulting in a total observation period of 21 days. In total, 604 high-quality hyperspectral image samples were collected for analysis. The study protocol targeted consistent longitudinal tracking. However, the final number of samples varied due to differences in participant availability and early termination of imaging after complete hematoma resolution. Each hyperspectral image has a spatial resolution of 512×512 pixels and contains 204 spectral channels spanning the wavelength range from 400 to 1000 nm. Figure 1 illustrates the temporal evolution of a hematoma for a representative participant, highlighting the early (0–3 days), middle (4–9 days), and late (10–20 days) stages.

All data partitioning was performed on a participant basis to prevent information leakage across time points [21]. A subset of participants was reserved during model development exclusively for hyperparameter tuning and convergence monitoring, while final performance evaluation relied solely on leave-one-subject-out (LOSO) cross-validation, as described in Section 2.4.

### 2.2. Preprocessing

**Figure 2:**
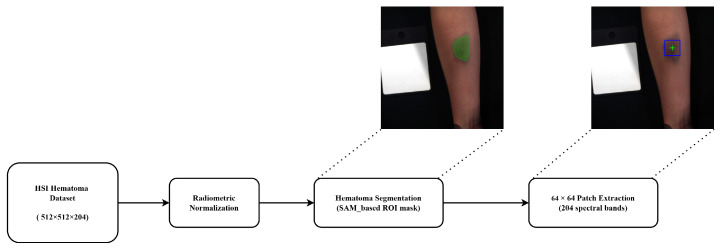
Preprocessing pipeline applied to the hyperspectral dataset. The workflow includes radiometric normalization, SAM-based hematoma segmentation (green shade), and patch extraction. The green cross denotes the center of the mask, while the blue box represents the extracted 64×64 pixel patch.

Processing hyperspectral images at their original resolution of 512×512×204 is computationally demanding and suboptimal for hematoma age estimation, as the region of interest occupies only a small portion of each image. As illustrated in Figure 1, large areas correspond to healthy skin or background, which may dilute hematoma-specific spectral information. To address this, a targeted preprocessing pipeline was designed to reduce computational complexity while emphasizing diagnostically relevant regions. The preprocessing workflow is illustrated in Figure 2 and consists of three main steps: radiometric normalization, hematoma segmentation, and patch extraction.

In the first step, radiometric normalization was applied to mitigate illumination variability and sensor-related effects by converting raw radiance values into relative reflectance [22,23]. The normalization was computed as

(1) $$I_{\mathrm{ref}}=\frac{I_{\mathrm{raw}}-I_{\mathrm{dark}}}{I_{\mathrm{white}}-I_{\mathrm{dark}}}$$

where $I_{\mathrm{raw}}$ denotes the raw hyperspectral image, and $I_{\mathrm{white}}$ and $I_{\mathrm{dark}}$ correspond to white and dark reference measurements, respectively. The resulting reflectance values were retained as 32-bit floating-point numbers (float32) without any quantization or binarization, preserving subtle spectral variations critical for discriminating hematoma stages.

**Figure 3:**
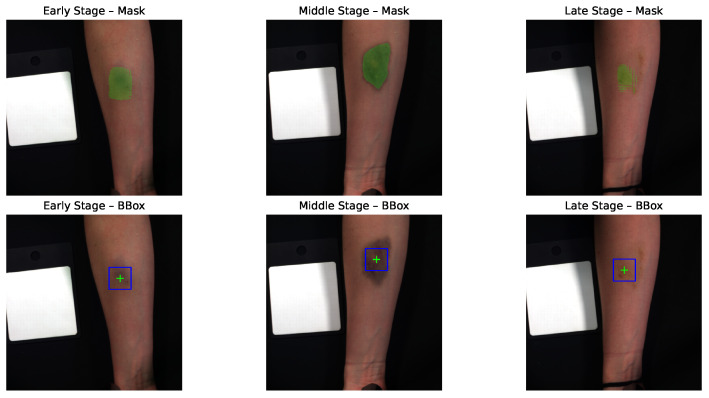
Segmentation examples for Participant 25, showing SAM-generated masks across early, middle, and late hematoma stages, together with the corresponding bounding boxes of size 64×64 used for patch extraction.

Hematoma segmentation was then performed using the Segment Anything Model (SAM) [24]. Although only a bounding box is ultimately required for patch extraction, segmentation was used as an intermediate step to accurately locate the hematoma center, ensuring that the extracted patches consistently covered the most affected region across time. This approach reduced manual annotation effort and improved reproducibility. The basic SAM model was chosen for its efficiency and sufficient accuracy for the task. To guide SAM toward the hematoma, a rough, manually defined bounding box was drawn around its approximate location. Samples without visible hematomas were excluded, particularly those captured shortly after blood injection (before hematoma formation) or at later stages when the hematoma had completely faded. This semi-automatic segmentation ensured that SAM-generated masks focused on the hematoma while minimizing inclusion of surrounding skin or background. Figure 3 shows examples of SAM masks and corresponding 64×64 bounding boxes centered on the segmented areas.

Finally, from each segmented region, a 64×64 patch was extracted while preserving all 204 spectral channels. This patch size balanced spatial coverage and computational efficiency: it was typically large enough to capture the central, most informative hematoma region, yet small enough to keep input dimensionality manageable. In some middle-stage samples, where the hematoma expanded, the entire hematoma did not fully fit within the patch. Conversely, in late-stage samples, as the hematoma began to fade and shrink, the extracted region also included a small portion of surrounding healthy skin. By consistently centering the extraction on the SAM-generated mask, the selected patches always captured the most diagnostically relevant region, whether this corresponded to the dense hematoma core in earlier stages or the residual discoloration in later stages. This ensured that each sample represented the most informative area for temporal hematoma evolution, while maintaining consistent input dimensions and reducing computational complexity.

### 2.3. Model Architectures

To evaluate the effectiveness of different modeling strategies for hematoma age estimation, we compared a simple linear baseline with a deep learning approach that jointly leverages spectral and spatial information. The two models differ in their ability to capture the underlying physiological patterns reflected in hyperspectral data.

#### 2.3.1. Baseline Model

Following the spectral-only analysis in Al-Arami et al. [20], the baseline model applies Lasso regression to the spatially averaged spectral signature. For each 64×64 patch, all pixel spectra were averaged, yielding a single 204-dimensional feature vector per sample. Lasso regularization was used to constrain model complexity and automatically select informative wavelengths, providing a simple and interpretable reference model that does not utilize spatial information.

#### 2.3.2. Proposed CNN Model

**Figure 4:**
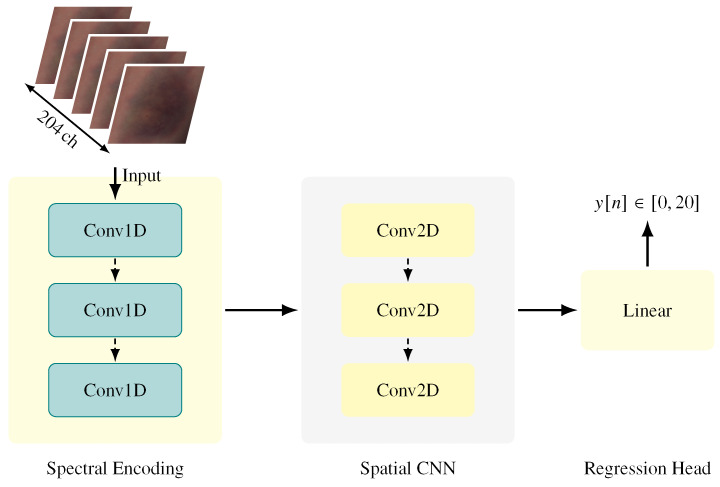
Overview of the proposed deep learning architecture for hyperspectral hematoma age estimation. The model employs a dual-stage feature extraction approach: three initial 1D convolutional layers isolate spectral signatures, followed by three 2D convolutional layers that capture the spatial morphology of the hematoma. The resulting feature maps are processed through a fully connected layer to perform the final regression of hematoma age (0–20 days).

To exploit both spectral and spatial cues, we designed a two-stage CNN that is illustrated in Figure 4. In the first stage, one-dimensional convolutions are applied along the spectral dimension to extract compact spectral embeddings that capture wavelength-dependent biochemical changes associated with hematoma maturation. In the second stage, two-dimensional convolutions operate on these embeddings to learn spatial patterns within the hematoma region, including distribution, shape, and localized absorption characteristics.

This hybrid spectral–spatial design explicitly models structured, wavelength-dependent relationships between channels while simultaneously capturing spatial heterogeneity within the hematoma region. Architectural details are summarized in Table 1.

### 2.4. Model Development and Evaluation

Model development followed a two-stage strategy comprising (i) hyperparameter tuning on a fixed participant-based split and (ii) final assessment with LOSO cross-validation.

During the tuning phase, a subset of participants was reserved exclusively for selecting model architectures and training parameters. Specifically, data from 19 participants were used for training, while data from three participants were used for validation. An additional three participants were held out as a tuning hold-out set to verify training stability. This split was employed solely for model selection and was not used for reporting final performance metrics.

Based on this phase, all models were trained with the Adam optimizer (learning rate $1\times 10^{-3}$, batch size 32, weight decay $1\times 10^{-5}$). To improve robustness and mitigate overfitting, random horizontal and vertical flips were applied as data augmentation. Training proceeded for up to 100 epochs with early stopping triggered if the validation Mean Absolute Error (MAE) failed to improve by at least 0.01 for 25 consecutive epochs.

To optimize training efficiency on the NVIDIA RTX A6000 GPU, we implemented Automatic Mixed Precision (AMP). Specifically, we utilized torch.autocast to dynamically select reduced precision (specifically float16) for compute-intensive operations during the forward and backward passes, while maintaining input data, model parameters, and loss accumulation in full precision (float32). To prevent arithmetic underflow often associated with reduced precision gradients, we employed gradient scaling (GradScaler) during backpropagation. This approach reduced memory overhead without altering the numerical representation of the source dataset.

Final model performance was assessed independently using a LOSO cross-validation protocol [25]. In each fold, data from one participant were held out for testing while the remaining 24 participants were used for training. This process was repeated until each participant served as the independent test subject exactly once. Performance metrics were averaged across all 25 folds and included MAE, Root Mean Square Error (RMSE), and the coefficient of determination ($R^{2}$). In addition, threshold accuracy was computed for error margins of ±1, ±2, and ±3 days to reflect increasingly strict temporal tolerance levels.

### 2.5. Spectral Configurations and Band-Importance Analysis

Beyond architectural design, we investigated how spectral resolution influences hematoma age estimation. Four input configurations were evaluated using the same CNN architecture:

(i) RGB Subset: A broad-band approximation containing bands at 460 nm, 540 nm, and 650 nm, simulating conventional color imaging.
(ii) Physiological Subset: An extended configuration including three additional hemoglobin-sensitive wavelengths of 504 nm, 569 nm, and 578 nm.
(iii) Full Range: The full hyperspectral range of 204 channels covering wavelengths of 400–1000 nm.
(iv) Top-20: A 20-channel configuration consisting of the twenty most informative spectral bands determined via feature attribution.

The selection of bands for configurations (i) and (ii) was driven by specific technical and physiological references. For the RGB Subset, the bands at 460 nm, 540 nm, and 650 nm were chosen specifically to align with the peak sensitivities of standard RGB Bayer filters, as established in [26]. Conversely, the additional bands in the Physiological Subset were selected to target the metabolic byproducts of heme degradation. Specifically, these bands capture the absorption maxima of bilirubin (≈460 nm), oxyhemoglobin (540 nm and 578 nm), and carboxyhemoglobin (≈569 nm), as detailed in forensic and clinical spectroscopy studies [27,28]. These subsets allow for a direct comparison between standard imaging and hyperspectral approaches while isolating physiologically motivated features.

The discrete bands for the Top-20 configuration were determined using a data-driven consensus of two feature-attribution techniques. First, a gradient-based saliency approach with noise averaging (SmoothGrad) [29,30] was used to estimate per-band relevance by computing the gradient of the output with respect to the input channels. Second, an occlusion-sensitivity method [31,32] was applied by sequentially masking each spectral band and measuring the resulting performance degradation. A consensus importance score was calculated by averaging the ranks from both methods, and the twenty bands with the highest scores were selected to form the subset.

## 3. Results

The proposed CNN demonstrated clear improvements over the baseline Lasso regression across all evaluation metrics. The results of the LOSO experiments are summarized in Table 2.

Specifically, the CNN reduced MAE from 3.24 to 2.29 days (−29.3%) and RMSE from 4.01 to 3.36 days (−16.2%), while increasing $R^{2}$ from 0.46 to 0.62. Furthermore, accuracy within ±1, ±2, and ±3 days increased by 19.4, 27.0, and 22.0 percentage points, respectively, indicating a substantially higher proportion of predictions within narrow temporal error bounds.

### 3.1. Comparison Baseline vs. Proposed CNN

**Figure 5:**
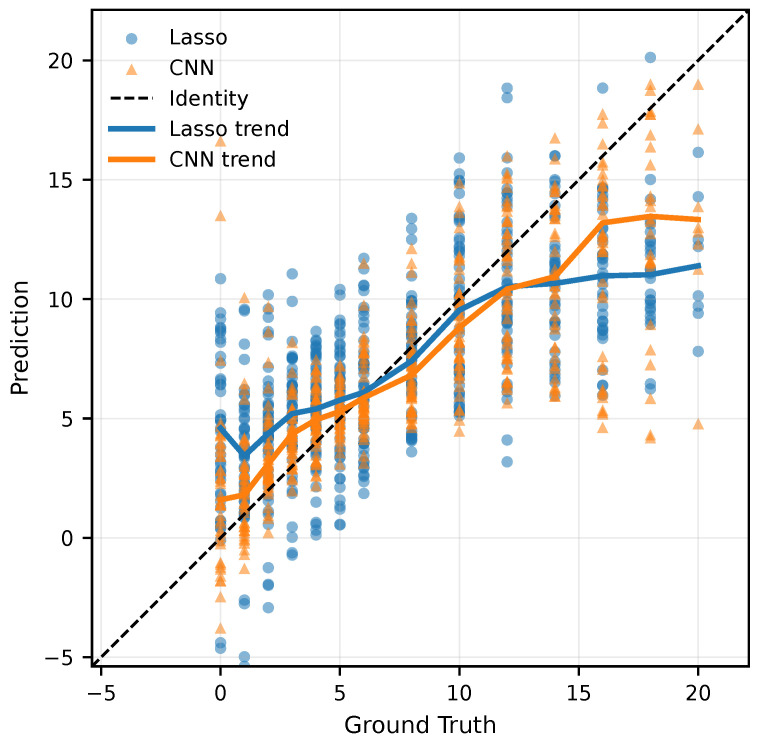
Scatter plot of predicted vs. ground truth hematoma ages (in days) for Lasso regression and the proposed Convolutional Neural Network (CNN). The CNN follows the identity line more closely and avoids the systematic underestimation observed in the baseline model.

To assess the added value of spatial information, the spectral-only Lasso regression baseline was compared with the proposed spectral–spatial CNN using the same LOSO evaluation scheme. Across all metrics, the CNN outperformed the baseline, showing lower error, better fit to the data, and a substantially higher proportion of predictions falling within clinically relevant error margins. The relationship between predicted and ground truth ages is illustrated in Figure 5. To visualize systematic bias across the 20-day age range, a locally weighted regression (LOWESS) trend line was applied to both models. This non-parametric smoothing technique provides an interpretable view of model behavior at different stages of healing. While both models track the identity line reasonably well in the mid-range, the Lasso baseline consistently underestimates the age of hematomas older than approximately 10 days. In contrast, the CNN remains closer to the identity line throughout the entire 20-day period. This demonstrates more stable predictive behavior across the early, middle, and late stages of healing.

**Figure 6:**
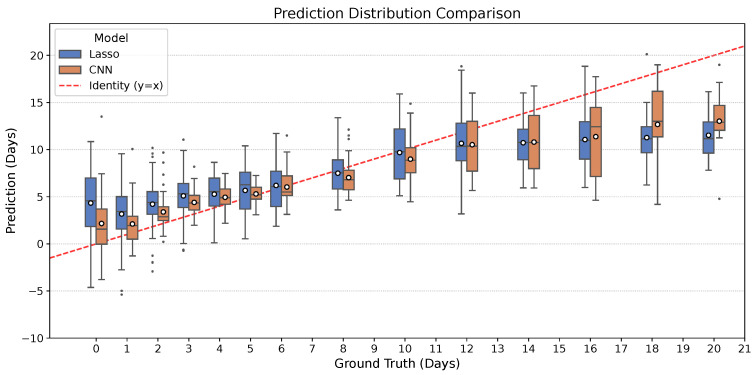
Prediction distribution comparison between the Lasso baseline and the proposed CNN. For each ground-truth age (0–21 days), the distribution of predictions is shown for both models. The red dashed line represents the ideal identity (y=x). The CNN (orange) displays tighter prediction distributions (narrower box plots) and central tendencies that align more closely with the ground truth compared to Lasso (blue), highlighting improved accuracy and reduced variability.

To further analyze prediction behavior across the healing timeline, Figure 6 summarizes the distribution of predicted ages for each ground-truth day (0–21 days). Compared to the Lasso baseline, the CNN consistently exhibits a narrower interquartile range and fewer extreme outliers, particularly in the early and late stages of healing. This visual reduction in spread indicates that the CNN provides significantly more stable predictions. Furthermore, the CNN’s mean predictions (indicated by white markers) generally track the ideal identity line (y=x) more closely than the baseline. These distributional trends complement the quantitative results in Table 2, demonstrating that the CNN not only achieves lower average error but also delivers better consistency across the entire trajectory.

### 3.2. Spectral Band Exploration and Importance Analysis

**Figure 7:**
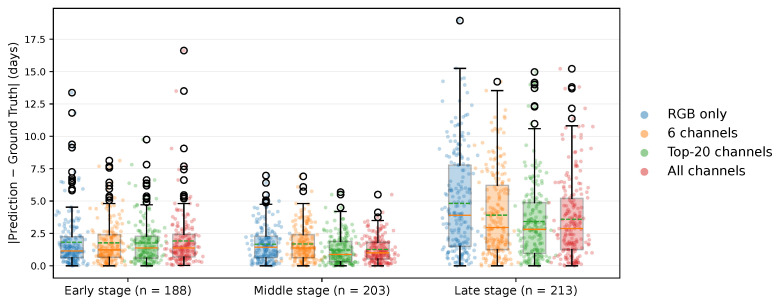
Absolute prediction errors for RGB-only, 6-channel, Top-20-channel, and full-band configurations across early (n=188), middle (n=203), and late (n=213) hematoma-healing stages (see Section 2.5). Within each boxplot, the solid orange horizontal line indicates the median, while the dashed green horizontal line represents the mean.

Model performance for the spectral configurations defined in Section 2.5 was analyzed across three temporal stages of hematoma healing, which were defined based on the dominant visual and biochemical phases: early (0–3 days), characterized by fresh hemoglobin and a reddish appearance; middle (4–9 days), representing the period of highest spectral contrast and stable chromophore coexistence; and late (10–20 days), characterized by bilirubin accumulation and fading, yellow-brown coloration. As shown in Figure 7, predicting hematoma age during the late healing stage was the most challenging across all input configurations.

The RGB-only configuration yielded the largest prediction errors (MAE = 4.82 days), followed by the six-channel hemoglobin-sensitive subset (MAE = 3.91 days) and the full 204-band configuration (MAE = 3.59 days), while the Top-20 subset achieved the lowest error (MAE = 3.42 days). In the middle stage, where hematomas are visually most pronounced, all methods performed well, but the Top-20 configuration achieved the best result (MAE = 1.22 days), slightly outperforming the full-band model (MAE = 1.26 days). In the early stage, all configurations produced similar MAEs between 1.7 and 1.9 days, whereas the Top-20 approach again performed best with MAE = 1.76 days.

Overall, the Top-20 subset matched or exceeded the performance of the full-band model in all stages, indicating that concentrating on a limited set of physiologically meaningful wavelengths can maintain or even improve predictive accuracy while substantially reducing data dimensionality.

**Figure 8:**
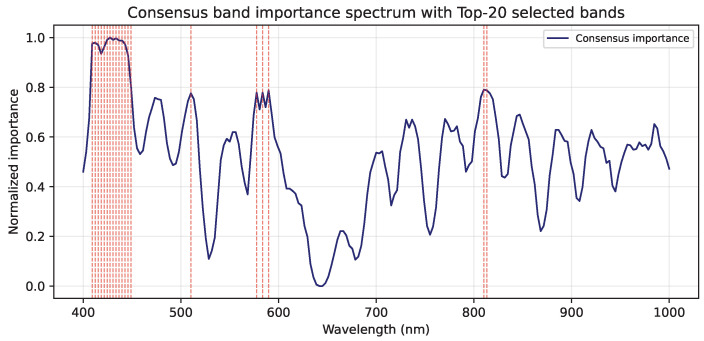
Consensus band-importance spectrum derived from SmoothGrad and occlusion sensitivity, highlighting the Top-20 selected bands (red).

Figure 8 shows the consensus band-importance spectrum derived from SmoothGrad and occlusion sensitivity.

The Top-20 most informative wavelengths (marked in red) are predominantly located in the Soret band region (405–445 nm), corresponding to hemoglobin absorption peaks that dominate during the early and middle hematoma stages. Additional clusters appear around 570–580 nm, associated with bilirubin and oxyhemoglobin absorption, and near 790–800 nm in the near-infrared range, reflecting sensitivity to deeper tissue scattering. These spectral patterns align well with known physiological absorption features and confirm that the CNN focuses on wavelength regions relevant to hematoma composition and evolution.

## 4. Discussion

The results of this study demonstrate that combining spectral and spatial information in a CNN substantially improves hematoma age estimation compared to a spectral-only Lasso regression baseline. Across all metrics, the CNN produced lower estimation errors, indicating that spatial heterogeneity within hematomas provides diagnostic information that is lost when spectral measurements are spatially averaged. In parallel, the finding that a compact Top-20 band configuration matched or exceeded the performance of the full 204-band input suggests that physiologically informed band selection can retain most of the relevant information while reducing sensor and computational complexity.

The observed performance gains can be attributed to physiologically grounded spectral characteristics of the tissue. Band-importance analysis showed that the CNN consistently emphasized wavelengths in the Soret band region (405–445 nm) and around 570–580 nm, which overlap with well-established absorption features of hemoglobin species and bilirubin reported in optical spectroscopy and diffuse reflectance studies [27,28]. These chromophores undergo systematic biochemical transformations during hematoma evolution, and the model’s sensitivity to these wavelength ranges is therefore consistent with known tissue optical properties and chromophore-specific absorption behavior [28]. Additionally, informative contributions observed around 790–800 nm likely reflect sensitivity to deeper tissue layers, where changes in scattering and hemodynamics are commonly assessed using near-infrared spectroscopy to probe blood volume and oxygenation [33]. Collectively, these findings indicate that the model leverages a physiologically meaningful subset of the spectrum rather than relying on spurious or dataset-specific correlations.

Model performance was not uniform across healing phases; the best results were obtained in the middle healing phase (4–9 days). During this interval, oxyhemoglobin and deoxyhemoglobin coexist in relatively stable proportions, generating strong spectral contrast against surrounding skin [34]. This richer biochemical signature likely facilitates more precise age estimation. In contrast, the weaker and more diffuse spectral characteristics of early and especially late stages make age estimation inherently more challenging, even with access to full hyperspectral information.

As hematomas progress toward resolution, their appearance becomes increasingly yellow-brown due to the accumulation of bilirubin and other hemoglobin degradation products [34,35]. Concurrently, the overall optical contrast diminishes, reducing spectral separability from normal skin. This effect is further complicated by melanin absorption, which contributes residual background signals and represents a particularly relevant confound for individuals with darker skin tones [36]. Moreover, late-stage hematomas tend to become spatially diffuse with poorly defined boundaries. In the present pipeline, this undermined RGB-based SAM segmentation and the subsequent patch extraction, leading to patches that may not fully capture faint or spatially extended spectral changes.

Several methodological limitations must be acknowledged. From a technical perspective, the current reliance on the SAM with a manually defined bounding box introduces sensitivity to the initial user input. The bounding box serves as a coarse spatial prompt that stabilizes segmentation in early and middle hematoma stages; however, in late-stage hematomas, where visual contrast is reduced, the accuracy of the resulting mask becomes increasingly dependent on prompt placement. This user dependence may affect reproducibility and is particularly pronounced for diffuse lesions whose spectral and spatial boundaries are poorly defined in RGB composites.

Processing full-resolution hyperspectral images (512×512×204) imposes substantial computational demands. To ensure tractable LOSO cross-validation, model training was therefore performed on 64×64 image patches centered on the hematoma region. Despite this dimensionality reduction, completing all 25 cross-validation folds required approximately 18 h on a single NVIDIA A6000 GPU. While the patch-based strategy enabled efficient training of the full-spectrum model, it necessarily restricted the spatial context available to the network, which likely limited its ability to capture the diffuse and extended characteristics of late-stage hematomas.

In terms of dataset characteristics, the study included 25 participants with predominantly light skin tones. The use of autologous blood injection provided high experimental control and a well-defined “time zero”; however, these experimentally induced hematomas do not fully reproduce the tissue trauma and variable lesion depth associated with naturally occurring bruises caused by blunt-force impact [34,37]. This constrains generalizability to real-world injuries, diverse populations, and other anatomical locations. Furthermore, the present results were obtained under controlled imaging conditions. In real-world forensic scenarios, such as crime scenes or post-mortem investigations, external factors including variable illumination and tissue decomposition, as well as physiological variations such as skin thickness and hemoglobin diffusion, may significantly influence the hyperspectral signal and introduce additional uncertainty [8,34,38].

Finally, the relatively small sample size combined with a high-capacity deep learning model raises the possibility of overfitting. The moderate predictive performance ($R^{2}=0.62$) suggests that while the model captures the underlying trends, the current dataset size is likely a limiting factor for achieving robust generalization. External validation on larger and more heterogeneous datasets is therefore required before clinical or forensic deployment.

## 5. Conclusions

This study demonstrates the feasibility and added value of hyperspectral imaging combined with deep learning for objective hematoma age estimation. In contrast to current forensic practice, which relies largely on subjective visual inspection and narrative plausibility, the proposed approach provides a quantitative alternative grounded in reproducible spectral and spatial evidence. By explicitly leveraging both spectral signatures and spatial heterogeneity, the convolutional neural network consistently outperformed a spectral-only regression baseline, underscoring the diagnostic relevance of spatially resolved hyperspectral information.

A key engineering outcome of this work is the identification of a consensus Top-20 subset of wavelengths that matched or exceeded the performance of the full 204-band configuration across all healing stages. This finding indicates that a physiologically informed reduction of spectral dimensionality can preserve the essential information required for hematoma age estimation while substantially reducing sensor complexity and computational cost. Notably, the reduced-band configuration decreased training time by approximately a factor of five (to 3.5 h), highlighting its practical relevance for scalable analysis and the future development of compact, multispectral imaging systems suitable for forensic deployment.

Future work should address both methodological and translational challenges. On the methodological side, developing fully automated lesion localization pipelines remains a critical objective. Replacing manually defined prompts with detector-based or hyperspectral-aware segmentation methods may improve robustness and reproducibility. However, achieving full automation for late-stage hematomas remains challenging due to the minimal spectral contrast between diffuse lesions and surrounding skin, suggesting that future pipelines may need to incorporate multispectral features beyond simple RGB composites to ensure feasibility.

In addition, the computational efficiency afforded by reduced-band acquisition may enable training on larger spatial regions or entire lesion masks, potentially mitigating the limitations of patch-based learning. Incorporating temporal or longitudinal modeling strategies, such as recurrent neural networks or transformer-based sequence architectures, also represents a promising direction for explicitly learning hematoma evolution trajectories rather than treating each acquisition independently.

From a translational perspective, expanding the dataset to include a broader range of skin tones, anatomical locations, and naturally occurring hematomas will be essential to improve generalizability and forensic utility. Although the standardized induction protocol used in this study (3 mL autologous blood) provided a well-defined ground truth and high experimental control, real-world injuries vary substantially in volume, depth, and mechanism. Consequently, external validation on independent cohorts and prospective studies of accidental or impact-related hematomas are required before routine forensic or clinical adoption can be considered.

While this research represents an early-stage investigation, the presented findings highlight the potential of hyperspectral imaging to support more objective and physiologically grounded hematoma assessment. In forensic settings, such a quantitative approach could complement expert judgment by providing reproducible evidence-based estimates. Beyond age estimation, hyperspectral analysis may facilitate standardized documentation of hematoma characteristics, including spatial heterogeneity and subtle temporal changes. In clinical contexts, objective HSI-based evaluation could assist in monitoring post-traumatic or postoperative hematoma progression and in identifying atypical healing patterns. Moreover, the demonstrated feasibility of reduced-band configurations makes this technology attractive for cost-sensitive applications, including animal welfare monitoring, where affordable and portable imaging solutions could enable improved detection and assessment of bruising.

## Figures and Tables

**Table 1:** Architectural summary of the proposed model.

Stage	Operation	Kernel/Params	Output Shape
Input	Hyperspectral cube	–	(204,64,64)
Spectral	Pixel-wise spectral reshape	–	(1,204)
Encoder	Conv1D	k=7, p=3	(128,204)
	Conv1D	k=5, p=2	(64,204)
	Conv1D	k=3, p=1	(32,204)
	AdaptiveAvgPool1D	L=16	(32,16)
	Spectral average	–	32 (per pixel)
	Spectral embedding map	–	(32,64,64)
Spatial	Conv2D	3×3	(64,64,64)
CNN	MaxPool2D	2×2	(64,32,32)
	Conv2D	3×3	(128,32,32)
	MaxPool2D	2×2	(128,16,16)
	AdaptiveAvgPool2D	4×4	(128,4,4)
Regression	Flatten	–	2048
Head	Linear	–	256
	Linear	–	1

Note: The spectral Conv1D encoder is applied independently to each pixel spectrum. All Conv layers are followed by ReLU and BatchNorm; dropout (0.3) is applied in the regression head.

**Table 2:** Quantitative performance comparison between the baseline and proposed models using leave-one-subject-out (LOSO) cross-validation.

Model	MAE [Days]	RMSE [Days]	$R^{2}$	Acc@1d [%]	Acc@2d [%]	Acc@3d [%]
Baseline (Lasso)	3.24	4.01	0.46	16.6	35.3	53.3
Proposed CNN	2.29	3.36	0.62	36.0	62.3	75.3

Values are reported as mean over 25 LOSO folds. Acc@Xd indicates the percentage of predictions within ±X days of the ground truth.

## Data Availability

The data presented in this study are available on request from the corresponding author due to ethical, privacy, and data ownership considerations.

## Abbreviations

The following abbreviations are used in this manuscript:

SAM	Segment Anything Model
CNN	Convolutional Neural Network
HSI	Hyperspectral Imaging
DRS	Diffuse Reflectance Spectroscopy
LOSO	Leave-One-Subject-Out
MAE	Mean Absolute Error
RMSE	Root Mean Square Error
AMP	Automatic Mixed Precision
